# Evaluation of intraocular pressure and corneal thickness in individuals at high altitude area (10000 ft above sea level)

**Published:** 2019

**Authors:** Arora Amit, Kapoor Gaurav, Ambiya Vikas, Kumar Ashok, A Singh Harpreet, Arora Shivani

**Affiliations:** * Base Hospital Delhi Cantt-110010, India; ** Army College of Medical Sciences, Delhi Cantt, India

**Keywords:** central corneal thickness, intraocular pressure

## Abstract

**Background.** At high altitude changes in corneal thickness and intraocular pressure (IOP) has been a subject of investigation for decades. The mechanism of action of these changes is still unknown. Extensive research is carried out to know effect of hypoxia on humans at high altitude. Corneal thickness and intraocular pressure are important parameter in ophthalmology with both diagnostic and therapeutic implications. We studied the corneal thickness and intraocular pressure in highlanders and compared the same with lowlanders.

**Methods.** This observational study included two groups, each consisting of 500 individuals residing at high altitude (more than 10,000 ft above sea level). Three measurements of intraocular pressure of both eyes in each group were noted with Goldmann applanation tonometer and mean of the three readings was taken. Central Corneal Thickness (CCT) was measured by ultra sound pachymetry. The parameters of two groups were compared statistically.

**Results.** There was a statistically significant difference between corneal thicknesses of the two groups studied, however no overall statistically significant difference in intraocular pressure of both these groups was found.

**Conclusion.** The highlanders have a thinner cornea as compared to lowlanders. The IOP is significantly higher in the highlander males as compared to lowlander males. However, the mean IOP is comparable between the overall population of the high altitude and that of low altitude.

## Background

At high altitude changes in corneal thickness and intraocular pressure (IOP) has been a subject of investigation for decades. High altitude may lead to a various systemic symptoms, including acute mountain sickness, high altitude pulmonary or cerebral edema. High altitude is usually regarded as an altitude over 2400m [**1**]. High altitudes lead to fall in atmospheric pressure as well as decrease in partial pressure of oxygen. The resultant hypobaric-hypoxia environment may be a contributing factor to the development of AMS [**2**]. Other contributing factors are ultraviolet rays, cold, and increased energy need. Extensive research is carried out to know effect of hypoxia on humans at high altitude. Corneal thickness is an important parameter in ophthalmology with both diagnostic and therapeutic implications. Corneal thickness influences intraocular pressure (IOP) readings as thicker corneas leading to overestimation of IOP and thinner, an underestimation of IOP thus affecting the glaucoma management [**3**]. Thinner corneas are an independent risk factor for the development of glaucoma in persons with ocular hypertension [**4**]. There are many factors that can affect corneal thickness, one of which is increasing altitude [**5**,**6**]. This is important as many people are travelling to higher altitudes. In 1918, Wilmer and Berens measured IOP in 14 aviators in a hypobaric chamber but found no signiﬁcant changes [**7**]. Various study groups have found different variations in IOP such as decrease in IOP [**8**], increased IOP [**9**,**10**], normal IOP [**11**,**12**], and even a reduction in IOP that occurred within hours of ascent and recovered during acclimatization [**13**,**14**]. The mechanism of these changes remains mysterious. It has also been suggested that aqueous humor dynamics and the relationship between intraocular and intracranial pressures are involved in these changes [**15**,**16**]. Various studies and case reports have highlighted an increase in corneal thickness in lowlanders on ascending to high altitude [**5**,**6**]. However, there are no studies available in the published literature about relationship of corneal thickness and intraocular pressure of the highlanders who are inhabitants of such altitudes. In this study, we studied the corneal thickness and intraocular pressure in highlanders and compared the same with lowlanders.

## Material & methods

This observational study included two groups, each consisting of 500 individuals residing at high altitude (more than 10,000 ft above sea level). Group I included subjects who were natural inhabitants at an altitude less than 3,000 feet, who were inducted by air for the first time to high altitude (more than 10,000 feet). Group II included natives of Leh district of Ladakh region of India, who were natural inhabitants at an altitude of more than 10,000 feet above sea level. The key inclusion criterion was age between 30 and 50 years. The key exclusion criteria were any pre-existing ocular disease (corneal pathologies, glaucoma, uveitis, or retinal disorders); past or current use of contact lens; currently on any systemic medication; presence of any systemic disease like hypertension/ diabetes). An informed consent was taken from each participant. Intraocular pressure (IOP) and corneal thickness of 500 eyes of group I were checked after 30 days of acclimatization at an altitude of 10,000 ft. Three measurements of intraocular pressure of both eyes in each group were noted with Goldmann applanation tonometer (GAT) and the mean of the three readings was taken. Central Corneal Thickness (CCT) was measured by using ultra sound pachymetry after using one drop of topical proparacaine hydrochloride 0.5% in the eye.

All measurements were taken between 10 am and 2 pm. A mean of 25 measurements of CCT of each eye of every participant was obtained and mean value was noted. The parameters of the two groups were compared.

## Results

**Table 1:** Comparison of CCT & IOP of Highlanders & Lowlanders

	Highlanders (n=500)	Lowlanders (n=500)	P
Age	35.58 ± 7.60	36.19 ± 7.42	0.20
M:F	290:210	398:102	<0.01
CCT (micron)	520.87 ± 50.21	548.06 ± 41.37	<0.01
IOP (mmHg)	17.84 ± 4.89	17.35 ± 4.17	0.09

**Table 2:** Comparison of CCT & IOP in gender matched Highlanders & Lowlanders

		Females (n=312)			Males (n=688)	P
	Highlander (n=210)	Lowlander (n=102)		Highlander (n=290)	Lowlander (n=398)	
Age	35.59 ± 8.59	36.22 ± 7.97	0.53	35.57 ± 6.78	36.19 ± 7.28	0.26
CCT (micron)	521.65 ± 51.79	551.08 ± 38.57	<0.01	520.38 ± 48.94	547.28 ± 42.07	<0.01
IOP (mmHg)	17.21 ± 4.79	17.57 ± 4.31	0.52	18.29 ± 4.91	17.29 ± 4.13	<0.01

In this study, IOP and corneal thickness of 500 highlanders and 500 lowlanders was evaluated. It was found that the mean intraocular pressure of lowlanders was 17.35 ± 4.17 mm Hg and for highlanders it was 17.84 ± 4.89 mm Hg (**Table 1**). Similarly, the corneal thickness of the same number of individuals was evaluated by ultrasound pachymetry. Overall, the mean corneal thickness of lowlanders was 548.06 ± 41.37 µm and for highlanders regardless of gender it was 520.87 ± 50.21 µm (**Table 1**). There was a statistically significant difference between the corneal thickness of the two groups studied, thinner corneas being found in highlanders as compared to lowlanders (**Table 1**). Intraocular pressure of highlander males (18.29 ± 4.91) was found to be higher statistically as compared to lowlander males (17.29 ± 4.13) (**Table 2**). However, no overall statistically significant difference in intraocular pressure of both these groups was found.

## Discussion

At high altitude, ambient temperature ad relative humidity is lower as comparison to low land. Another potential hazard is high solar ultraviolet radiation in environment. There is 10% increase in ultraviolet radiation with every 1000 m altitude ascent [**4**]. This is further enhanced by climatic factors such as terrain, latitude, altitude, sun elevation, ground reﬂection, and ozone. So, at high altitude the eye is exposed to higher UV radiation than at plains. Prolonged exposures to solar UV radiation may result in short-term and long-term health effects on various human organs and their functions [**17**].

Corneal thickness is affected by corneal metabolism, endothelial function, as well as oxygen tension. Morris et al. found that central corneal thickness (CCT) increased signiﬁcantly on increasing altitude in healthy corneas of group of lowlanders [**18**].

Liu HM et al. have done systematic review and meta analysis of central corneal thickness of Healthy Lowlanders at High Altitude and found that significant increase in central corneal thickness with ascent to altitude and decrease in CCT after descent [**19**]. Patyal S et al. studied corneal thickness in highlanders. They studied central corneal thickness of 254 highlanders and comparing it with the one of 212 lowlanders; they found thinner corneas in highlanders as compared to lowlanders and difference was significant [**20**].

Various studies results in literature regarding central corneal thickness are contradictory. We studied central corneal thickness in highlanders who are permanent natives and compared with lowlanders. We found that there is a statistically significant difference in CCT of highlanders, being thinner cornea as compared to lowlanders. Effect of high altitude on Intraocular pressure (IOP) has been a subject of investigation. Various studies suggested that controversial relationship between intraocular, intracranial pressures and aqueous humour dynamics [**15**,**16**]. Non-pigmented ciliary epithelium secretes aqueous by both active and passive mechanisms. Various systemic factors such as blood pressure, IOP, and the venous osmotic pressure affect ultrafiltration and diffusion. Aqueous drainage is influenced by rise in episcleral venous pressure. Ascent to altitude produces a characteristic physiologic response, which may alter IOP due to vasogenic cerebral edema [**21**], vascular endothelial damage [**22**], change in cerebrovascular autoregulation [**23**], sustained vasodilatation, and raised cerebral capillary pressure [**24**]. A recent study has reported a statistically significant rise in IOP during ascent to high altitude [**25**]. Ersanli et al. concluded small rise in IOP in heathy individuals after ascent to high altitude, they also concluded that this small rise in IOP seen in the healthy subjects might be much greater in older persons or those with limits on aqueous drainage, such as preexisting ocular hypertension or glaucoma. They also concluded that with oxygen masks, intraocular pressures can rise to significant levels in climbers with ocular hypertension or glaucoma [**26**]. Pavlidis M et al. studied a direct proportional correlation between decreases in pO2 and IOP. It is found that blood pressure increased during acclimatization with decrease in IOP. Every new active exposure to hypobaric hypoxia in the ascent phase induced a decrease in the IOP parallel to the decrease in pO2. This suggests that IOP changes are related to hypoxia and acclimatization stage [**27**]. 

We studied intraocular pressure and corneal thickness in 500 highlanders and 500 lowlanders. Intraocular pressure difference in both these populations was statistically not significant. However, significantly higher IOP was found as compared to lowlanders among male population of highlanders. Central corneal thickness of both populations showed statistically significant difference, thinner cornea being found in highlanders as compared with lowlanders.

## Conclusion

The highlanders have a thinner cornea as compared to lowlanders. The IOP is significantly higher in the highlander males as compared to lowlander males. However, the mean IOP is comparable between the overall population of the high altitude and that of low altitude. 

**Conflict of interest**

None.

## References

[1] Muza SR, Beidleman BA, Fulco CS (2010). Altitude preexposure recommendations for inducing acclimatization. High Alt Med Biol.

[2] Emergency and Continuous Exposure Guidance Levels for Selected Submarine Contaminants.

[3] Rand R, Allingham KF, Damji S (2005). Shield’s Textbook of Glaucoma.

[4] Medeiros FA, Weinreb RN (2012). Is corneal thickness an independent risk factor for glaucoma?. Ophthalmology.

[5] Morris DS, Somner JEA, Scott KM (2007). Corneal thickness at High altitude. Cornea.

[6] Jha KN (2012). High Altitude and the eye. Asia Pac J Ophthalmol (Phila).

[7] Wilmer WH, Berens C (1918). The effect of altitude on ocular functions. JAMA.

[8] Brinchmann-Hansen O, Myhre K (1989). Blood pressure, intraocular pres- sure and retinal vessels after high altitude mountain exposure. Aviat Space Environ Med.

[9] Ersanli D, Yildiz S, Sonmez M, Akin A, Sen A, Uzun G (2006). Intraocular pressure at a simulated altitude of 9000 m with and without 100% oxygen. Aviat Space Environ Med.

[10] Carapancea M (1977). Experimental and clinical hyperophthalmotony of high altitudes. Arch Ophthalmol Rev Gen Ophthalmol.

[11] Clarke C, Duff J (1976). Mountain sickness, retinal haemorrhages and acclimatisation on Mount Everest in 1975. BMJ.

[12] Bayer A, Yumusak E, Sahin OF (2004). Intraocular pressure measured at ground level and 10,000 feet. Aviat Space Environ Med.

[13] Cymerman A, Rock PB, Muza SR (2000). Intraocular pressure and acclimatization to 4300 M altitude. Aviat Space Environ Med.

[14] Pavlidis M, Stupp T, Georgalas I (2006). Intraocular pressure changes during high-altitude acclimatization. Graefes Arch Clin Exp Ophthalmol.

[15] Lashutka MK, Chandra A, Murray HN, Phillips GS, Hiestand BC (2004). The relationship of intraocular pressure to intracranial pressure. Ann Emerg Med.

[16] Jonas JB, Berenshtein E, Holbach L (2004). Lamina cribrosa thickness and spatial relationships between intraocular space and cerebrospinal ﬂuid space in highly myopic eyes. Invest Ophthalmol Vis Sci.

[17] World Health Organization (2002). Global Solar UV Index (A Practical Guide).

[18] Morris DS, Somner JEA, Scott KM (2007). Corneal thickness at high altitude. Cornea.

[19] Liu HM , Bai CH , Liou CM , Chiou HY, Chen C (2018). Central Corneal Thickness of Healthy Lowlanders at High Altitude: A Systematic Review and Meta-Analysis. Curr Eye Res.

[20] Patyal S, Arora A, Yadav A, Sharma VK (2017). Corneal Thickness in Highlanders. High Alt Med Biol.

[21] Bartsch P, Roach RC (2001). Acute mountain sickness and high-altitude cerebral edema. In: Hornbein TF, Schoene RB, eds., High Altitude: An Exploration of Human Adaptation.

[22] Schilling L, Wahl M (1999). Mediators of cerebral edema. In: Roach RC, Wagner PD, Hackett PH, eds. Hypoxia: into the Next Millennium.

[23] Levine BD, Zhang R, Roach RC (1999). Dynamic cerebral autoregulation at high altitude. In: Roach RC, Wagner PD, Hackett PH, eds. Hypoxia: into the Next Millennium.

[24] Hackett PH, Roach RC (2001). High-altitude illness. N Engl J Med.

[25] Somner JEA, Morris DS, Scott KM (2007). What happens to intraocular pressure at high altitude?. Invest Ophthalmol Vis Sci.

[26] Ersanli D, Yildiz S, Sonmez M, Akin A, Sen A, Uzun G (2006). Intraocular pressure at a simulated altitude of 9000 m with and without 100% oxygen. Aviat Space Environ Med.

[27] Pavlidis M (2006). Intraocular pressure changes during high-altitude acclimatization. Graefes Arch Clin Exp Ophthalmol.

